# The Intrinsically Disordered C-Terminal Domain Triggers Nucleolar Localization and Function Switch of PARN in Response to DNA Damage

**DOI:** 10.3390/cells8080836

**Published:** 2019-08-05

**Authors:** Tian-Li Duan, Guang-Jun He, Li-Dan Hu, Yong-Bin Yan

**Affiliations:** State Key Laboratory of Membrane Biology, School of Life Sciences, Tsinghua University, Beijing 100084, China

**Keywords:** DNA damage response, function switch, intrinsically disordered domain, nucleolar localization, phosphorylation, poly(A)-specific ribonuclease (PARN), RNA maturation, structure switch

## Abstract

Poly(A)-specific ribonuclease (PARN), a multifunctional multi-domain deadenylase, is crucial to the regulation of mRNA turnover and the maturation of various non-coding RNAs. Despite extensive studies of the well-folding domains responsible for PARN catalysis, the structure and function of the C-terminal domain (CTD) remains elusive. PARN is a cytoplasm–nucleus shuttle protein with concentrated nucleolar distribution. Here, we identify the nuclear and nucleolar localization signals in the CTD of PARN. Spectroscopic studies indicated that PARN-CTD is intrinsically disordered with loosely packed local structures/tertiary structure. Phosphorylation-mimic mutation S557D disrupted the local structure and facilitated the binding of the CTD with the well-folded domains, with no impact on PARN deadenylase activity. Under normal conditions, the nucleolus-residing PARN recruited CBP80 into the nucleoli to repress its deadenylase activity, while DNA damage-induced phosphorylation of PARN-S557 expelled CBP80 from the nucleoli to discharge activity inhibition and attracted nucleoplasm-located CstF-50 into the nucleoli to activate deadenylation. The structure switch-induced function switch of PARN reshaped the profile of small nuclear non-coding RNAs to respond to DNA damage. Our findings highlight that the structure switch of the CTD induced by posttranslational modifications redefines the subset of binding partners, and thereby the RNA targets in the nucleoli.

## 1. Introduction

In eukaryotic cells, the length of the non-templated oligo(A) or poly(A) tail at the 3′-end of RNAs is regulated by the opposing actions of polyadenylation and deadenylation [1]. Modulation of oligo(A) or poly(A) tail length is crucial to almost all RNA turnover processes including processing, maturation, transportation, localization, function and degradation [2,3,4,5,6]. Particularly, removal of the poly(A) tail is the rate-limiting step of the decay of most mRNAs via the deadenylation-dependent degradation pathway [7]. Poly(A) tail length regulation also plays a regulatory role in translational control [8,9,10,11,12]. Deadenylation is achieved by deadenylases, which are a group of Mg^2+^-dependent 3′–5′ exonucleases with high substrate preference of oligo(A) or poly(A) [2,4]. Deadenylases are also involved in the 3′-end trimming process of small RNAs [13,14,15,16]. By modulating RNA metabolism, deadenylases have been identified to participate into diverse biological processes [2,4,17,18,19,20].

Most eukaryotic cells possess a number of deadenylases, although all of them have the same catalytic function of removing adenosines from the RNA 3′-end [2,4]. Every deadenylase generally targets not all, but a distinct subset of RNAs during a certain cellular process. This specificity may be determined by their diverse catalytic properties, multiple subcellular distributions, numerous binding partners and various post-translational modifications [2]. Among the deadenylases identified thus far, poly(A)-specific ribonuclease (PARN, EC. 3.1.13.4) is unique for its high catalytic activity, processive degradation of poly(A), nucleolar localization and ability to bind with both the 5’-end cap and 3′-end poly(A) tail simultaneously [21]. These properties are endowed by the multi-domain dimeric structure, which is comprised of a catalytic DEDD domain, a R3H RNA-binding domain, a RNA recognition motif (RRM) and a long C-terminal extension (C-terminal domain, CTD). Previous extensive structural and biochemical studies have shown that the three substrate binding domains (DEDD, R3H and RRM) work synergistically to recognize the poly(A) substrate and degrade it processively (reviewed in [21]). To precisely regulate the poly(A) tails of a subset of transcripts, PARN can be recruited by a number of RNA-binding proteins [2,21].

One of the most defined functions of PARN is its complicated actions in DNA damage response (DDR). Under normal conditions, PARN can direct the decay of p21 and p53 mRNAs, and thus modulate cell cycle progression [18,22]. During DDR, p53 protein is proposed to bind and activate PARN to degrade transcripts involved in the p53-dependent DDR pathway [22]. In p53-defective tumor cells, the DNA damage-activated MAPKAP kinase-2 (MK2) phosphorylates PARN at Ser557 to block Gadd45αmRNA degradation [23]. Meanwhile, CBP80-inhibited PARN can be activated by CstF-50 under DNA damaging conditions [24,25]. PARN may also modulate DDR by affecting the RNA levels of key genes involved in telomere biology [26,27]. Finally, PARN contributes to the 3′-end formation of non-coding RNAs involved in DDR, such as human Y RNA [28] and some miRNAs [29]. The above findings suggest that alterations in the PARN phosphorylation status and protein-protein interaction network are two important aspects to sophisticatedly modify the fates of key RNAs in response to DNA damage. An unresolved problem is whether PARN phosphorylation affects its binding with various partners.

MK2 phosphorylates PARN at Ser557, which locates on the CTD. Unlike the well-studied RNA-binding domains, little is known about the structure and function of the CTD. The CTD has been predicted to be intrinsically disordered based on in silico analysis of the amino acid sequence [30,31]. Previous biochemical and biophysical analysis indicates that the CTD contributes little to PARN structural integrity and catalysis [32,33], while playing a role in PARN stability and self-association [34]. The cytoplasm localization of proteolytic fragments of *Xenopus* PARN, which lacks the CTD, and human PARN, which lacks the CTD as well as half of the RRM, implies that the CTD contains the nuclear localization sequence (NLS) [35,36]. Although it has been observed that PARN is abundant in nucleoli and Cajal bodies in human cells [13], it remains unclear whether PARN possesses the nucleolar localization sequence (NoLS). An interesting question is whether phosphorylation of S557, which is located at the CTD, modifies the subcellular localization of PARN. In this research, we identified the NLS and NoLS in PARN-CTD. Furthermore, we verified that the CTD is an intrinsically disordered domain by spectroscopic experiments, and found that PARN-CTD contributed to protein–protein interactions. PARN phosphorylation resulted in the recruitment of different binding partners into the nucleoli during DDR to switch its RNAs targets, and thereby affect cell survival upon genotoxic stresses. Our results highlight that the diverse functions of PARN are probably endowed by the multiple sets of binding partners recruited by the CTD. Post-translational modifications of the CTD redefine the protein–protein interaction network, and thereby switch PARN function.

## 2. Materials and Methods

### 2.1. Materials

Tris and diethypyrocarbonate were purchased from Amresco (Solon, OH, USA). Mouse anti-Flag-tag monoclonal antibody, anti-Flag M2-agrarose, protease inhibitor cocktail, leupeptin, phenylmethylsulfonyl fluoride (PMSF), Triton X-100, Tween-20, Nonidet P40 (NP-40), hydroxyl urea (HU), ribonuclease A (RNase A), bovine serum albumin (BSA), paraformaldehyde, imidazole, sodium dodecyl sulfate (SDS), guanidine hydrochloride (GdnHCl), polyadenylic acid potassium salts (catalog number: P9403-25MG), DTT, kanamycin, ampicillin and isopropyl-1-thio-β-D-galactopyranoside (IPTG) were obtained from Sigma-Aldrich (St. Louis, MO, USA). Rabbit anti-PARN and mouse anti-nucleophosmin (NPM1) antibodies were from Abcam (Cambridge, MA, USA). FITC AffiniPure goat anti-mouse IgG, FITC AffiniPure goat anti-rabbit IgG, mouse anti-HA, rabbit anti-HA, DyLight 594 AffiniPure goat anti-mouse, DyLight 594 AffiniPure goat anti-rabbit, HRP AffiniPure goat anti-mouse and HRP AffiniPure goat anti-rabbit antibodies were from EarthOx (Millbrae, CA, USA). The Annexin V-FITC/PI apoptosis detection Kit was obtained from Bioworld (Louis Park, MN, USA). The transfection reagent VigoFect was from Vigorous (Beijing, China). Hoechst 33342 was from Invitrogen (Carlsbad, CA, USA). All other reagents were local products of analytical grade.

### 2.2. Plasmid Construction, Protein Expression and Purification

The plasmid containing the cDNA sequence of the wild type (WT) human PARN was kindly gifted by Professor Anders Virtanen (Uppsala University). The PARN-CTD fragment (amino acid residues 541–639) was constructed using the following primers: PARN-CTD-forward (F), 5′-GATGTCACCATATGCCCCAGTGCATACCCTA-3′, PARN-CTD-reverse (R), 5′-TGACCTCGAGTTACCATGTGTCAGGAAC-3′. The genes were cloned to the vector pET-28a (Novagen, Madison, WI, USA) and verified by sequencing. Details regarding the purification of recombinant proteins overexpressed in *Escherichia coli* Rosetta (DE3) were the same as those described previously with some modifications [33,37]. In brief, the overexpression of the recombinant proteins was induced by 0.025 mM IPTG at 10 °C for 48 h. The His-tagged proteins were extracted from cell lysates by Ni^2+^-affinity chromatography and purified using a Superdex 200 16/60 pre-grade column. The purity of the final products was above 98%, as estimated by SDS-PAGE. The protein concentration was determined by the Bradford method using BSA as the standard [38]. The purified proteins were concentrated in buffer A containing 20 mM Tris-HCl, pH 8.0, 100 mM KCl, 0.5 mM DTT, 0.2 mM EDTA and 20% (*v*/*v*) glycerol, aliquoted and stored at −80 °C. The fully denatured protein was obtained by denaturing the protein in buffer A with the addition of 6 M GdnHCl at room temperature overnight.

### 2.3. Size-Exclusion Chromatography Analysis

Size-exclusion chromatography (SEC) analysis was performed on an ÄKTA purifier equipped with a Superdex 75 10/300 GL column (GE Healthcare, Madison, WI, USA). The column was pre-equilibrated for 2 column volumes using buffer A with a flow rate of 0.4 mL/min. The protein solutions pre-equilibrated at 4 °C were loaded on the injection ring with a volume of 100 μL. The UV absorbances at 280, 254 and 215 nm were monitored simultaneously during the flow of the samples.

### 2.4. Spectroscopy

The spectroscopic experiments were carried out at room temperature using a protein concentration of 0.2 mg/mL in buffer A. Far-UV circular dichroism (CD) spectra were measured on a Jasco-715 spectrophotometer using a cell with a pathlength of 0.1 cm and a spectral resolution of 0.2 nm. The percentages of various secondary structure elements were analyzed by CDPro using the CONTIN, SELCON, and CDSSTR methods [39] and the presented data were average ± SD of the three methods. CD signals were expressed as mean residue molar ellipticity ([*θ*_MRW_]). Intrinsic fluorescence spectra excited at 295 or 280 nm were measured on a Hitachi F-2500 spectrophotometer using a 0.2 mL cuvette, with a slit width of 5 nm for both excitation and emission and a spectral resolution of 0.5 nm. The intrinsic fluorescence excited at 295 nm is dominated by Trp fluorophores and was used for the extraction of various classes of fluorophores by the discrete state model [40,41] using house-made software, as described previously [42].

### 2.5. Enzyme Assay

Deadenylase activity of PARN was measured using the SEC method as described elsewhere [43]. In brief, the SEC experiments were performed by a Superdex 200 10/300 GL column (GE Healthcare, Madison, WI, USA), which was pre-equilibrated with 2 column volumes of the reaction buffer in the absence of MgCl_2_. The standard reaction buffer contained 20 mM Tris-HCl (pH 7.0), 100 mM KCl, 1.5 mM MgCl_2_, 0.5 mM DTT, 0.2 mM EDTA and 10% (*v*/*v*) glycerol. The reaction was initiated by mixing 20 µL enzyme and 100 µL substrate stock solutions. The reaction was carried out at 37 °C and quenched on ice, and then the quenched samples were analyzed by SEC. The concentration of the produced AMP was determined according to the standard curve.

### 2.6. Cell Culture

Human embryonic kidney (HEK)-293T, Chinese-hamster ovary (CHO) and HeLa cell lines were obtained from ATCC. The cells were cultured in the Dulbecco’s modified Eagle’s medium (DMEM, Gibco, Grand Island, NY, USA) with the addition of 10% fetal bovine serum (FBS, Gibco) at 37 °C in a humidified incubator with 5% CO_2_. DNA damaging treatment of the cells was performed by exposing the cells to 100 J/m^2^ UV light for 10 min or culturing the cells in DMEM medium with the addition of 1 mM HU for 3 h. Then, the treated cells were transferred to fresh DMEM medium and cultured for 2 h before further analysis.

### 2.7. Immunofluorescence Microscopy

The targeted genes were subcloned to the pEGFP-C3 or pEGFP-N1 expression vector, which were used to exogenously express proteins fused with EGFP at the N-terminus or C-terminus, respectively. The CHO or HEK-293T cells were seeded on glass coverslips and transfected with the plasmid by VigoFect according to the standard protocol provided by the manufacturer. After 24 h of cultivation, the cells were washed with phosphate-buffered saline (PBS) twice, fixed by 4% paraformaldehyde for 1 h, permeabilized with 0.4% Triton X-100 for 30 min and then blocked with 10% goat serum for 1 h. Immunostaining was carried out using the primary antibody at 4 °C overnight. The sample was then washed with PBS three times and incubated with the corresponding secondary antibody for 1 h at room temperature. The nuclei were counterstained with Hoechst 33342. Images were obtained using a Carl Zess LSM 710 confocal microscope system (Carl Zeiss, Jena, Germany).

### 2.8. Co-Immunoprecipitation (Co-IP) Assay

The sequences encoding full-length or fragmented PARN were subcloned into pcDNA3.1 (Invitrogen) with the inclusion of a Flag tag, while those encoding CBP80 or CstF-50 were subcloned with a HA tag. Prior to transfection, HEK-293T cells were seeded in a 60 mm dish and cultured for 24 h to reach 60–80% confluence. The plasmids containing Flag-PARN and HA-CBP80/HA-CstF-50 were co-transfected with a plasmid concentration of 5 μg using the Vigofect transfection reagent, according to the manufacturer’s instructions. After 24 h cultivation, the HEK-293T cells were washed with ice-cold PBS and harvested by scraper and centrifugation. After freezing at –80°C, the harvested cells were lysed by 200 µL IP lysis buffer containing 50 mM Tris-HCl (pH 7.4), 150 mM NaCl, 1 mM EDTA, 1 mM ethyleneglycoltetraacetic acid (EGTA), 1 mM NaF, 1% NP-40, 1 mM PMSF and 1 mg/mL leupeptin. The cell lysates were centrifuged for 20 min at 15,000 g at 4 °C. A 40 µL aliquot of supernatant was taken as the total cell lysate (TCL). The remaining supernatant was incubated with 20 µL of anti-Flag M2-agarose beads overnight on ice. Then the beads were washed three times gently with the IP lysis buffer. Both the supernatants and pellets were examined by Western blot using anti-Flag or anti-HA antibodies.

### 2.9. Cell Apoptosis Assay

The HEK-293T cells transfected with plasmids containing the WT or fragmented PARN sequence were cultured in the DMEM medium for 24 h and then the medium was removed. The medium-free cells were exposed to 100 J/m^2^ UV light for 10 min, transferred and cultivated in fresh DMEM medium for 2 h, harvested, washed with cold PBS and resuspended with the buffer containing Annexin V-FITC and propidium iodide (PI) according to the manufacturer’s instructions. Cell apoptosis was analyzed using a FACS Calibur flow cytometer (BD Bioscience, San Diego, CA, USA).

### 2.10. Real-Time PCR

The total RNA was extracted from the cells using a TIANGEN RNA extraction kit (TIANGEN Biotech Co., Ltd., Beijing, China). A 2 µg portion of the total RNA was reverse-transcribed using TIANScript M-MLV RT Kit (TIANGEN) according to the manufacturer’s instructions. The obtained cDNA was used for real-time PCR analysis using LightCycler480 II (Roche, Basel, Switzerland) and SYBR Green dye (Invitrogen, Carlsbad, CA, USA). The forward (F) and reverse (R) primers used for real-time PCR analysis were as follows: p21-F, 5′-TGAGCCGCGACTGTGATG-3′; p21-R, 5′-GTCTCGGTGACAAAGTCGAAGTT-3′; PPIA-F, 5′-GTCAACCCCACCGTGTTCTT-3′; PPIA-R, 5′-CTGCTGTCTTTGGGACCTTGT-3′; RPLP0-F, 5′-GGCGACCTGGAAGTCCAACT-3′; RPLP0-R, 5′-CCATCAGCACCACAGCCTTC-3′; SNORA9-F, 5′-CAAGCCTCCAGCGTGCTTG-3′; SNORA9-R, 5′-CATTGTCTGAAATTTCTATAACC-3′; SCARNA8-F, 5′-GGAGGCTGATACACAAATTG-3′; SCARNA8-R, 5′-GTATCTGTCCGTTACGATTTC-3′.

### 2.11. Statistical Analysis

Statistical analysis was performed using GraphPad Prism software (GraphPad Software Inc., San Diego, CA, USA). The unpaired two-tailed Student’s *t*-test was used to compare the set of data assuming a Gaussian distribution and a *p* value less than 0.05 was considered significant. All quantitative data were obtained from three repetitions.

## 3. Results

### 3.1. PARN-CTD Is Intrinsically Disordered with Loosely Packed Local Structures

**Figure 1:**
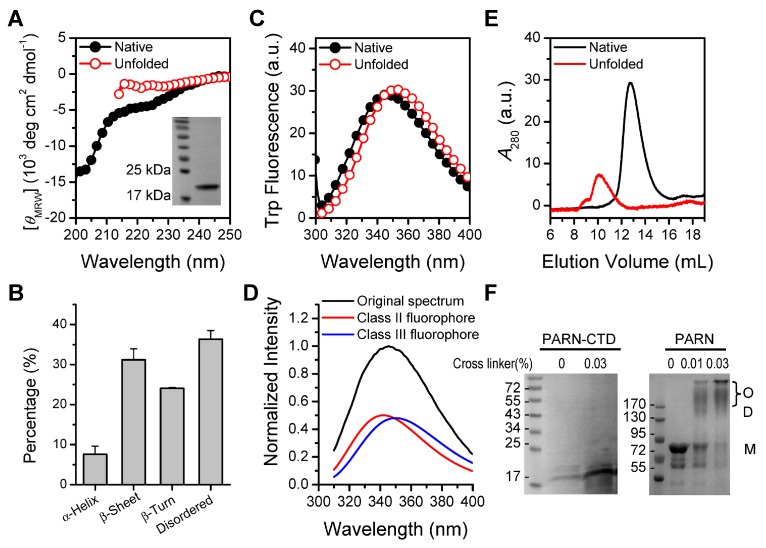
Structural features of poly(A)-specific ribonuclease C-terminal domain (PARN-CTD)**.** (**A**) Far-UV CD spectra of native PARN-CTD(541–639) and the unfolded state fully denatured by 6 M GdnHCl. The inset shows the SDS-PAGE analysis of the purified recombinant protein. (**B**) Percentages of various secondary structure elements estimated by CDPro. (**C**) Intrinsic Trp fluorescence excited at 295 nm. (**D**) Fitting of the experimental fluorescence spectrum of native PARN-CTD by the discrete state model of Trp residues in proteins. (**E**) SEC profiles of the native and unfolded proteins. (**F**) Analysis of the purified PARN-CTD by cross-linking using 0.03% glutaraldehyde by SDS-PAGE. Cross-linking of the full-length PARN, which exists as a dimer and high-order oligomers in solutions [49], was used as a positive control. M, monomer; D, dimer; O, oligomer. The molecular weight of the marker proteins are shown in kDa.

Despite the extensive structural studies of the nuclease domain, R3H domain and RRM [44,45,46,47], little is known about the structural properties of PARN-CTD. Although previous bioinformatics analysis has suggested that the CTD might be intrinsically disordered [30,31], no experimental evidence is available yet. To gain insight into the structural features of the CTD, we screened the expression and purification conditions of several fragments of the CTD and successfully purified a little PARN-CTD (amino acids 541–639) using a low IPTG concentration of 0.06 mM and a low incubation temperature of 10 °C for 48 h. The structural features were studied by biophysical techniques via comparing with the 6 M GdnHCl-denatured protein. The theoretical molecular weight of PARN-CTD with a His-tag at the N-terminus is 13.2 kDa and the molecular weight of the purified protein was verified by mass spectrometry analysis. The far-UV CD spectrum of the native PARN-CTD revealed a large negative peak below 203 nm and a minor negative peak at around 225 nm (Figure 1A). The pattern of the CD spectrum of native PARN-CTD was consistent with a protein dominated by disordered structures and was similar to the distinctive shape of the other intrinsically disordered proteins [48]. Analysis of the percentages of secondary structural elements by CDpro [39] indicated that PARN-CTD contained a small content of regular structures (Figure 1B). The existence of regular secondary structures was evidenced by the divergence between the CD spectrum of the native protein and that of the fully denatured form (Figure 1A).

PARN-CTD contained only one Trp fluorophore, W639, located at the C-terminus. The intrinsic fluorescence spectrum of PARN-CTD (Figure 1C) had an emission maximum wavelength (*E*_max_) of 345 nm, which is 7 nm away from the fully denatured form (352 nm). This suggested that, compared with the fully denatured state, W639 in PARN-CTD was partially protected by local structures. Fitting the spectrum by the theoretical model of discrete states of Trp residues in proteins [40,41,42], the Trp fluorescence of PARN-CTD contained about half of the Class II component, centered at 340 nm, and half of the Class III component, centered at 352 nm (Figure 1D). Classes II and III represent Trp fluorophores that are exposed to bound waters or are fully water-exposed, respectively [40]. Since PARN-CTD contained only one Trp residue, the existence of two types of fluorophores implied that PARN-CTD could adopt two distinct states with similar Gibbs free energy or stability. However, there was only one dominant peak in the SEC profile (Figure 1E), suggesting that there was a fast conformational exchange between the structured and disordered states.

Native PARN-CTD eluted at around 12.8 mL, which was much earlier than the fully denatured state (10.1 mL) and later than lysozyme, which has a molecular weight of 14 kDa (17.2 mL). The smaller hydrodynamic radius of the native PARN-CTD is consistent with the above proposal that PARN-CTD was not in a completely extended disordered state, but in a state with local structures. The apparent molecular weight of PARN-CTD determined by SEC was calculated to be around 22 kDa, which is much larger than that of a monomer (13.2 kDa). The large apparent molecular weight was probably caused by the larger hydrodynamic volume of intrinsically disordered proteins, since cross-linking experiments indicated that PARN-CTD was a monomer in solution (Figure 1F).

Environmental factors may modulate the structural transition of intrinsically disordered proteins from unstructured to structured [48]. We investigated the effects of various factors, including temperature, pH, K^+^ concentration, divalent metal ions, low concentrations of denaturants and macromolecular crowding reagent, and representative results are shown in Figure S1. None of the screened conditions significantly modified the structure of PARN-CTD, as revealed by the almost superimposed far-UV CD spectra and unchanged *E*_max_ of Trp fluorescence. Most factors affect the fluorescence intensity in a non-specific way, except for K^+^, which showed an unusual enhancement in a K^+^ concentration-dependent manner with a *K*_d_ value of 7 mM (Figure S2). This phenomenon was similar to that observed in full length PARN [34].

### 3.2. Identification of NLS and NoLS in the CTD of PARN

**Figure 2:**
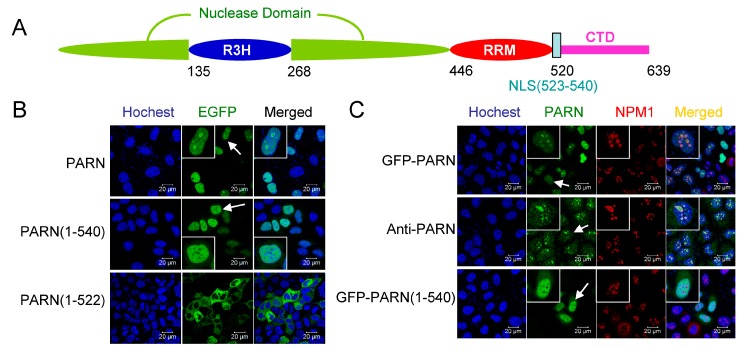
Identification of the nuclear localization sequence (NLS) in PARN. (**A**) A schematic model of domain compositions of human PARN. The position of the NLS is labeled. (**B**) Verification of the putative PARN-NLS(523–540) by confocal microscopy analysis of Chinese-hamster ovary (CHO) cells transiently transfected with plasmids containing EGFP-fused PARN and the two truncated forms. The represented images show the results of EGFP fused to the N-terminus of PARN, and similar results were obtained for EGFP fused to the C-terminus of PARN (data not shown). The nuclei were counterstained with Hoechst 33342. (**C**) PARN-NLS(523–540) directed the nucleoplasm but not nucleoli localization of PARN. The nucleoli were marked by nucleophosmin (NPM1) using mouse anti-NPM1 antibody (red). Endogenous PARN was immunostained by rabbit anti-PARN antibody (green). In panels B and C, the scale bars represent 20 µm and the insets show the magnified region indicated by the arrows.

One potential function of the CTD is to mediate the nuclear localization of PARN. Previous studies have shown that PARN is a nucleus-cytoplasm shuttle protein with pronounced distribution in the nucleoli [13]. In *Xenopus*, the full-length PARN mainly exists in the nucleus while its proteolytic fragment, p62, localizes in the cytoplasm [35]. The PORT II prediction algorithm [50] revealed no NLS in human PARN, but that amino acid residues 522–539 were a putative bipartite NLS for *Xenopus* PARN. Sequence alignment indicated that the putative NLS of *Xenopus* PARN was conserved in vertebrates, corresponding to amino acid residues 523–540 in human PARN (Figure 2A and Figure S3A).

To verify the putative NLS, GFP fused PARN and its truncated mutant were exogenously expressed in the CHO cells by transient transfection. The results in Figure 2B clearly show that the region 523–540 was responsible to the nuclear localization of PARN. Furthermore, both of the exogenously expressed GFP-PARN and endogenous PARN stained by mouse anti-PARN antibody were enriched in the nucleoli (Figure 2C), similar to that observed previously [13]. For the cell lines studied here, the distribution of PARN in Cajal bodies was not pronounced, which might be caused by the limited number of Cajal bodies in these cells. The fact that PARN(1–540) containing NLS (523–540) mainly distributed in the nucleoplasm, but not the nucleoli, indicated that there was addition sequence(s) or binding partner(s) responsible for the nucleolar localization of PARN.

**Figure 3:**
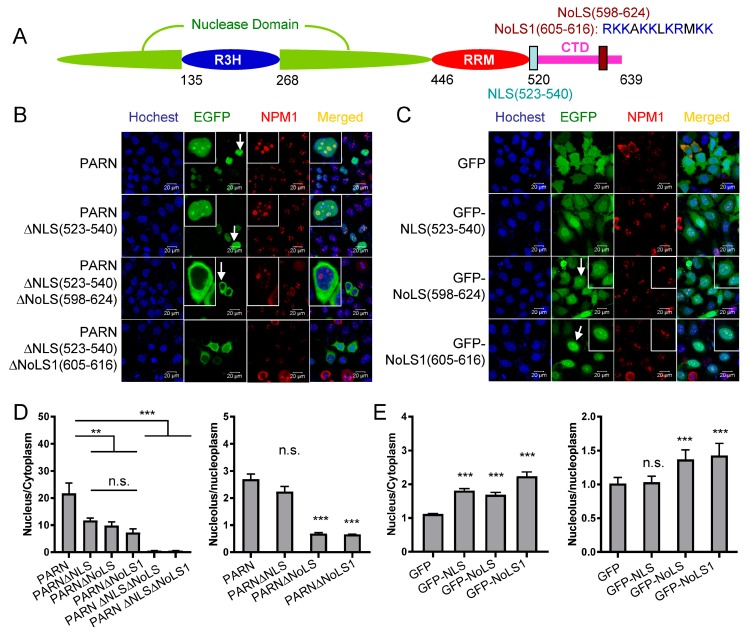
Identification of nucleolar localization sequence (NoLS) in PARN. (**A**) The position and core sequence of the NoLS are labeled in the schematic model of PARN domain compositions. NoLS1 represents a shorter sequence than the predicted NoLS, and contains the positively charged residue cluster. (**B**) Verification of PARN-NLS and -NoLS by single or double deletion mutants. (**C**) Verification of PARN-NLS and -NoLS by fusing the corresponding sequences to the C-terminus of EGFP. (**D**) Quantitative analysis of the relative nuclear (left) and nucleolar (right) distributions of PARN and various deletion mutants. The relative distribution ratio was calculated from the mean fluorescence intensities of the corresponding subcellular compartments. (**E**) Quantitative analysis of the relative nuclear (left) and nucleolar (right) distribution of GFP fused with NLS or NoLS. In panels B and C, the scale bars represent 20 µm and the insets show the magnified region indicated by the arrows. ** *p* < 0.01; *** *p* < 0.001; n.s., not significant.

To distinguish whether PARN itself contains the NoLS or is recruited to the nucleoli by its binding partner, the putative NoLS in PARN-CTD was analyzed by the NoD web server [51] and a potential NoLS was found between amino acid residues 598 and 624. Sequence alignment (Figure S3B) indicated that this region was conserved among vertebrates and the core motif of positively charged residues was located at 605–616, which was designated as NoLS1 (Figure 3A). Confocal microscopy analysis indicated that although the nuclear distribution of PARN was weakened by the removal of the NLS, PARNΔNLS retained the ability to localize in the nucleoli (Figure 3B,C). Removal of either NoLS or NoLS1 significantly reduced both the nuclear and nucleolar distributions of PARN. Removal of both of NLS and NoLS completely blocked PARN from entering the nucleus. Meanwhile, GFP fused with either NLS or NoLS greatly enhanced its distribution in the nucleus (Figure 2D,E). GFP-NLS did not concentrate in the nucleoli, while GFP fused with NoLS or NoLS1 had pronounced enrichment in the nucleoli. The above results clearly indicated that either NLS or NoLS could direct PARN to enter the nucleus, while the core sequence in NoLS was indispensable for the nucleolar localization of PARN.

### 3.3. The CTD Contributes to PARN Function in DDR by Modulating the Protein–Protein Interaction Network

**Figure 4:**
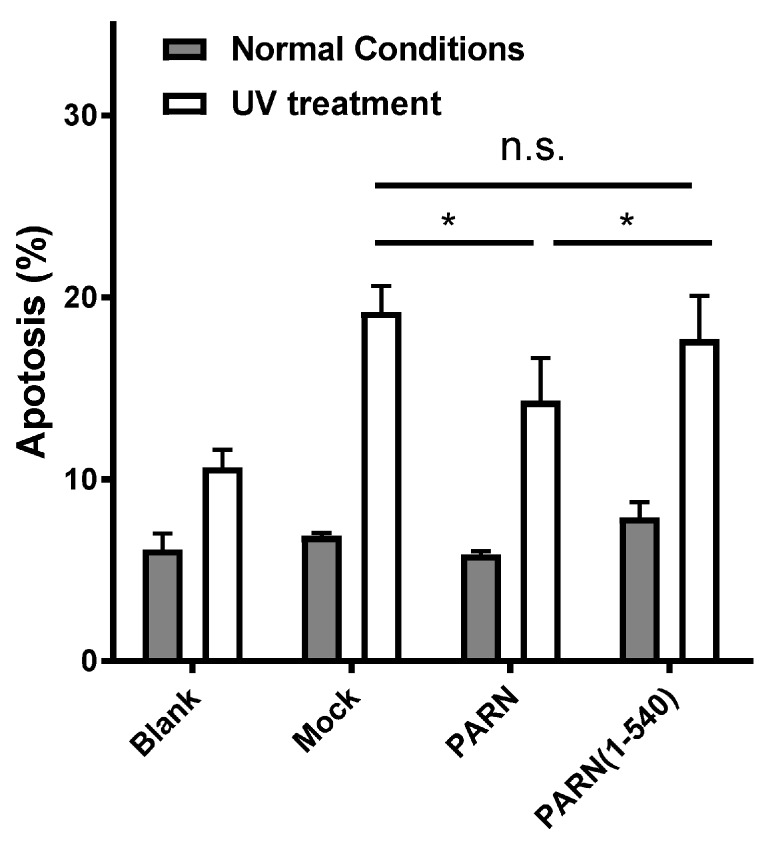
Effect of exogenously expressed PARN on cell apoptosis induced by UV treatment. Percentages of apoptotic HEK-293T cells were analyzed by flow cytometry, as seen in Figure S4. Data are presented as mean ± SD calculated from at least three independent experiments. * *p* < 0.05; n.s., not significant.

To gain insight into the role of the CTD in DDR, the HEK-293T cells were transiently transfected with plasmids containing Flag-tagged full length PARN and the truncated form PARN(1–540). Due to the complicated effects of PARN depletion under DNA-damaging conditions [18], overexpression, but not rescue, experiments were used in this study to avoid potential unexpected variables introduced by PARN knockdown. The effect of exogenously expressed proteins on cell survival was analyzed by bivariate flow cytometry using Annexin V-FITC and PI staining. Under normal conditions, no significant effect was observed for the exogenously expressed PARN and its mutant (Figure 4 and Figure S4). Under UV-treated conditions, the exogenously expressed PARN could enhance cell survival by slightly decreasing the percentage of apoptotic cells. The removal of the CTD abolished this beneficial effect of PARN on cell survival. This result is consistent with the previous observation that knockdown of endogenous PARN promotes apoptosis or necrosis of gastric cancer cells [18].

**Figure 5:**
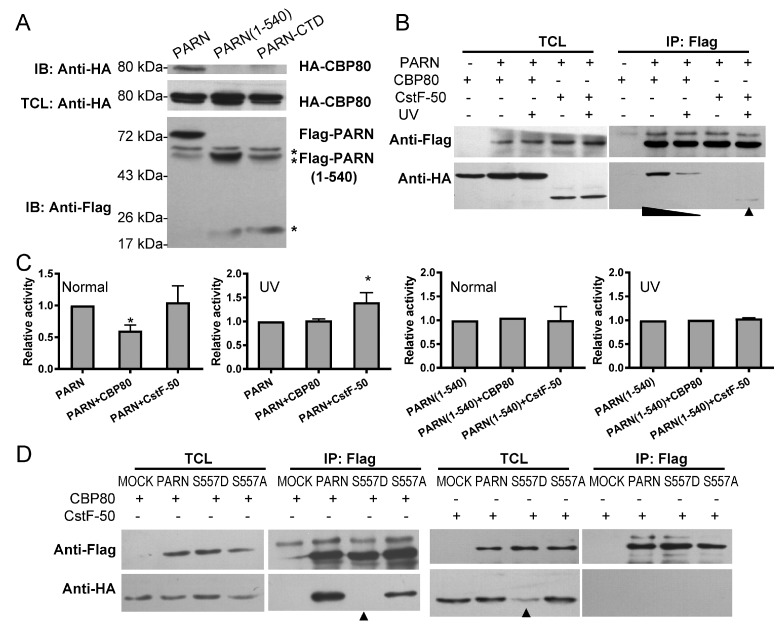
The CTD contributes to the interactions of PARN with CBP80 and CstF-50. (**A**) Co-IP experiments using the anti-Flag antibody to detect the interaction between HA-CBP80 and Flag-PARN. IB represents immunoblot using anti-Flag antibody to detect Flag-PARN and anti-HA antibody to detect the HA-CBP80. TCL is the total cell lysates of the HEK-293T cells. The asterisks indicate the Ig heavy and light chains. The band of PARN(1–540) overlapped with that of the Ig heavy chain. (**B**) Co-IP experiments to identify the interactions between Flag-PARN and HA-CBP80/ HA-CstF50 in untreated and UV-treated HEK-293T cells. (**C**) Effect of CBP80 or CstF-50 on the deadenylase activity of PARN in TCL of the HEK-293T cells (*n* = 3). The deadenylation activity of PARN was determined by the SEC method. The amounts of PARN expression level were detected by the western-blot and adjusted to the same level for all testing groups. (**D**) Effect of S557D mutation on the binding of Flag-PARN with HA-CBP80 and HA-CstF-50. MOCK is the control group of cells co-transfected with HA-CBP80/HA-CstF-50 and the empty vector. * *p* < 0.05.

PARN may participate in DDR via multiple mechanisms occurring in both the nucleus and the cytoplasm. Consistent with previous observations [18,22], we also found that the exogenously expressed PARN could regulate the protein level of p53 (Figure S5). However, most of the pathways identified thus far either occur in the cytoplasm or are achieved by accelerating the degradation of specific subsets of RNAs via the deadenylase activity of PARN. These actions are probably not through the CTD. Among the various pathways, the activation of the CBP80-inactivated PARN by CstF-50 is an event that occurs in the nucleus [24,25]. Previous studies have shown that the C-terminal fragment of PARN(443–639), containing both the RRM and CTD, is essential for PARN-CstF-50 interaction [24]. To elucidate the contributing domain in mediating protein–protein interactions, Co-IP experiments were carried out in HEK-293T cells transiently co-transfected with plasmids containing Flag-PARN and HA-CBP80/HA-CstF-50. All assayed samples were treated by RNAse A to avoid indirect interactions induced by the bound RNAs. Consistent with the previous results [24,25], HA-tagged CBP80 was co-precipitated with the full-length PARN (Figure 5A). PARN(1–540), which lacks the CTD, could not pull down CBP80, indicating that the CTD was indispensable for the interaction between PARN and CBP80.

Unfortunately, the expression of PARN-CTD was undetectable in the transfected HEK-293T cells since the isolated CTD was susceptible to degradation due to its intrinsically disordered property. Under normal conditions, no interaction between PARN and CstF-50 could be detected (data not shown, see also Figure 7). When the cells were treated by UV irradiation, the amount of co-precipitated HA-CBP80 greatly decreased, accompanied with the appearance of a small amount of co-precipitated HA-CstF-50 (Figure 5B). Co-transfection of CBP80 reduced PARN deadenylase activity in lysates of the untreated cells, while co-transfection of CstF-50 enhanced the activity under UV-treated conditions (Figure 5B). Co-transfection of CBP80 or CstF-50 did not influence PARN(1–540) activity, confirming that the CTD mediated PARN inactivation by CBP80 and activation by CstF-50.

A possible pathway to switch PARN-binding partners during DDR is through post-translational modifications of the CTD. Under DNA damaging conditions, a known post-translational modification of PARN is phosphorylation at S557 by MK2 [23]. Phosphorylation-induced folding has been proposed to act as a regulatory switch of intrinsically disordered proteins [52]. We also verified the previous results that PARN-S557 was one of the phosphorylated sites under UV-irradiated conditions in the HEK-293T cells by mass spectrometry (data not shown). To elucidate the role of S557 phosphorylation in protein–protein interactions, PARN-S557D and PARN-S557A mutants were constructed to mimic the phosphorylated and unphosphorylated forms. As shown in Figure 5D, the S557D mutation completely abolishes the ability of PARN to interact with CBP80 under normal conditions, whereas the PARN-S557A mutant behaves the same as the WT PARN. Unexpectedly, both PARN-S557D and PARN-S557A mutants could not pull down detectable amounts of CstF-50. It is worth noting that the expression level of HA-CstF-50 in cells co-transfected with plasmids containing Flag-PARN-S557D was much lower than the other groups. Consistently, the exogenously expressed PARN greatly reduced the protein level of endogenous CstF-50 in UV-treated cells (Figure S5). The down-regulation of the CstF-50 protein level by PARN was not directed by the CTD, since PARN(1–540) had the same effect. Further research is needed to study the underlying mechanism and functional implications of CstF-50 down-regulation by PARN.

**Figure 6:**
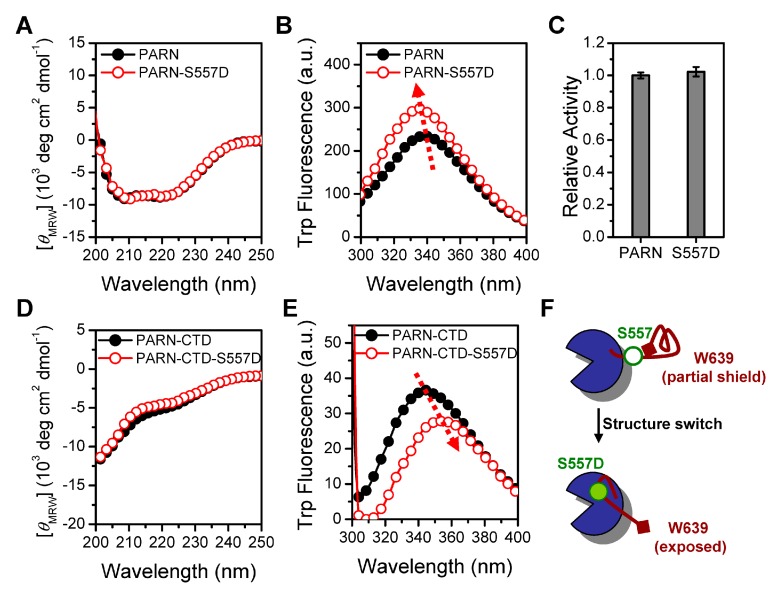
Phosphorylation-mimic S557D mutation induces structure switch of PARN-CTD. (**A**) far-UV CD spectra of PARN and PARN-S557D. (**B**) Trp fluorescence spectra of PARN and PARN-S557D. (**C**) Effect of S557D on the deadenylase activity of PARN. (**D**) Far-UV CD spectra of PARN-CTD(541–639) and PARN-CTD(541–639)-S557D. (**E**) Trp fluorescence spectra of PARN-CTD(541–639) and PARN-CTD(541–639)-S557D. (**F**) A schematic model of the structure switch induced by the S557D mutation. The S557mutation disrupts the local structure shielding the W639 fluorophore and facilitates the binding of CTD with the main body of PARN.

To gain insight into the structural basis of the functional switch induced by S557 phosphorylation, we purified phosphorylation-mimic recombinant proteins PARN-S557D and PARN-CTD-S557D. This mutation did not affect PARN deadenylase activity (Figure 6).

The almost superimposed far-UV CD spectra indicated that the S557D mutation had no impact on protein secondary structures. However, the mutation induced an opposing effect on the tertiary structures of PARN-S557D and PARN-CTD-S557D when monitored by Trp fluorescence. The S557D mutation blue-shifted the *E*_max_ of PARN from 340 to 335 nm, accompanied by a significant increase in Trp fluorescence intensity, while a red-shift of *E*_max_ from 346 to 353 nm and a decrease of Trp fluorescence intensity was observed in PARN-CTD. In the full-length protein, the S557D-induced blue shift and intensity increase of Trp fluorescence implied that the mutation facilitated the Trp fluorophores in PARN to bury in more solvent-inaccessible or hydrophobic microenvironments. The extra shielding of the Trp fluorophores could have arisen from tight local structure formed around W639 or microenvironmental changes around the other Trp fluorophores in the main body of PARN. The *E*_max_ of PARN-CTD-S557D was the same as the fully denatured PARN-CTD protein (Figure 1B), suggesting that this mutation led the partial shielded fluorophore W639 to a fully water-exposed state. Thus, it is more likely that the S557D mutation disrupted the local structure around W639 in the CTD. The lack of local structure promoted the CTD to interact with the main body of the molecule, and thereby influenced the microenvironments around the Trp fluorophores in the main body (Figure 6F). Although the S557D mutation did not alter the intrinsically disordered nature of the CTD, the different extents of loosely packed local structures might prefer to interact with dissimilar binding partners.

### 3.4. DNA Damage-Induced Phosphorylation Redefines PARN-Binding Partners in the Nucleoli to Reshape the Profile of Small Nuclear RNAs

**Figure 7:**
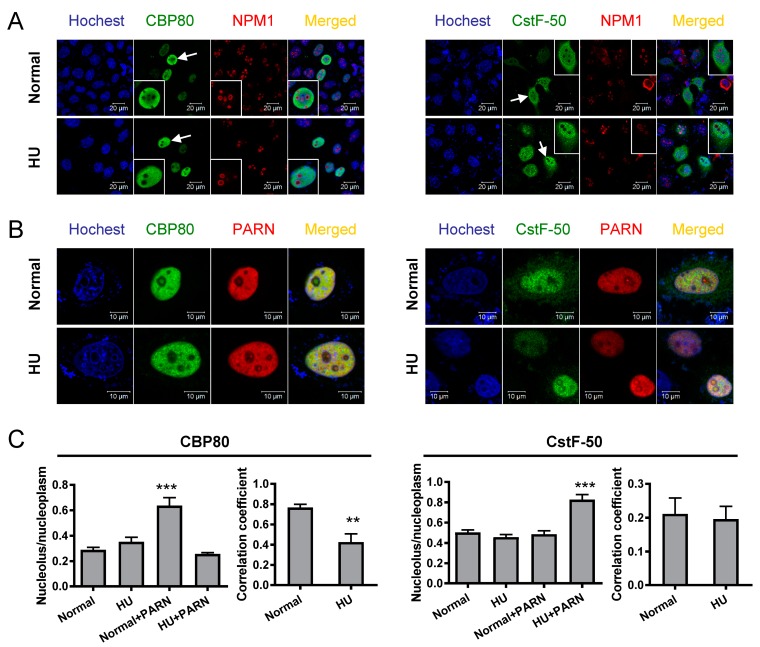
The CTD triggers recruitment of CBP80/CstF-50 into the nucleoli by PARN. (**A**) Subcellular distributions of HA-CBP80 and HA-CstF-50 (green) in untreated and 1 mM HU-treated CHO cells. The nucleoli were marked by mouse anti-NPM1 antibody (red). The nuclei were counterstained with Hoechst 33342. (**B**) Colocalization analysis of HA-CBP80/HA-CstF-50 and Flag-PARN in untreated and HU-treated CHO cells. The scale bar represents 20 µm. (**C**) Quantitative analysis of the relative distribution of CBP80 or CstF-50 in the nucleoli and the Pearson’s correlation coefficient of PARN- CBP80/CstF-50 co-localization (*n* = 10). ** *p* < 0.01; *** *p* < 0.001.

The above results indicated that the CTD contributed to both nucleolar localization and protein–protein interactions of PARN. The functional relationship between these two events was studied by the subcellular localization of PARN and CBP80/CstF-50 in the CHO cells under both normal and DNA damaging conditions (Figure 7). During DDR, no significant change was observed for the nucleolar localization of PARN. The two mutations, S557D and S557A, did not affect the subcellular localization of PARN (data not shown). A mild reagent, HU, which inhibits DNA synthesis to mimic DNA damage, was used to facilitate confocal microscopy studies. CBP80 mainly concentrated in the nucleoplasm, but not nucleoli, under both normal and HU-treated conditions. CstF-50 had both nuclear and cytoplasm distributions under normal conditions, while the nuclear fraction was increased when the cells were treated by HU. The fact that both CBP80 and CstF-50 had little distribution in the nucleoli suggested that they themselves did not possess the NoLS. Pronounced nucleolar distribution could be identified for CBP80 in the untreated cells and CstF-50 in the HU-treated cells when the cells were co-transfected with PARN. Pearson’s correlation coefficient [53] was used to quantitatively characterize the colocalization of CBP80/CstF-50 and PARN. Under normal conditions, the Pearson’s correlation coefficient of PARN-CBP80 was around 0.7. This large value implied that PARN was able to recruit CBP80 into the nucleoli. Under DNA damaging conditions, CBP80 was expelled from the nucleoli, probably due to the phosphorylation of PARN-S557. The Pearson’s correlation coefficient between PARN and CstF-50 was around 0.2, much lower than that between PARN and CBP80 in the untreated cells. It is worth noting that the nucleolar localization of CstF-50 was only obvious in cells with high CstF-50 protein levels (Figure 7B, right panel). Thus, the poor PARN-CstF-50 colocalization was probably caused by the down-regulation of CstF-50 expression by PARN under DNA damaging conditions, coinciding with the co-IP results shown in Figure 5. It is also worth noting that the exogenously expressed CBP80 and CstF-50 also weakened the nucleolar localization of PARN when comparing the results seen in Figure 7 with those in Figure 3, probably due to the alteration in CBP80 and CstF-50 levels restricting more PARN molecules in the nucleoplasm and affecting the nucleoplasm-nucleoli shuttle of PARN.

**Figure 8:**
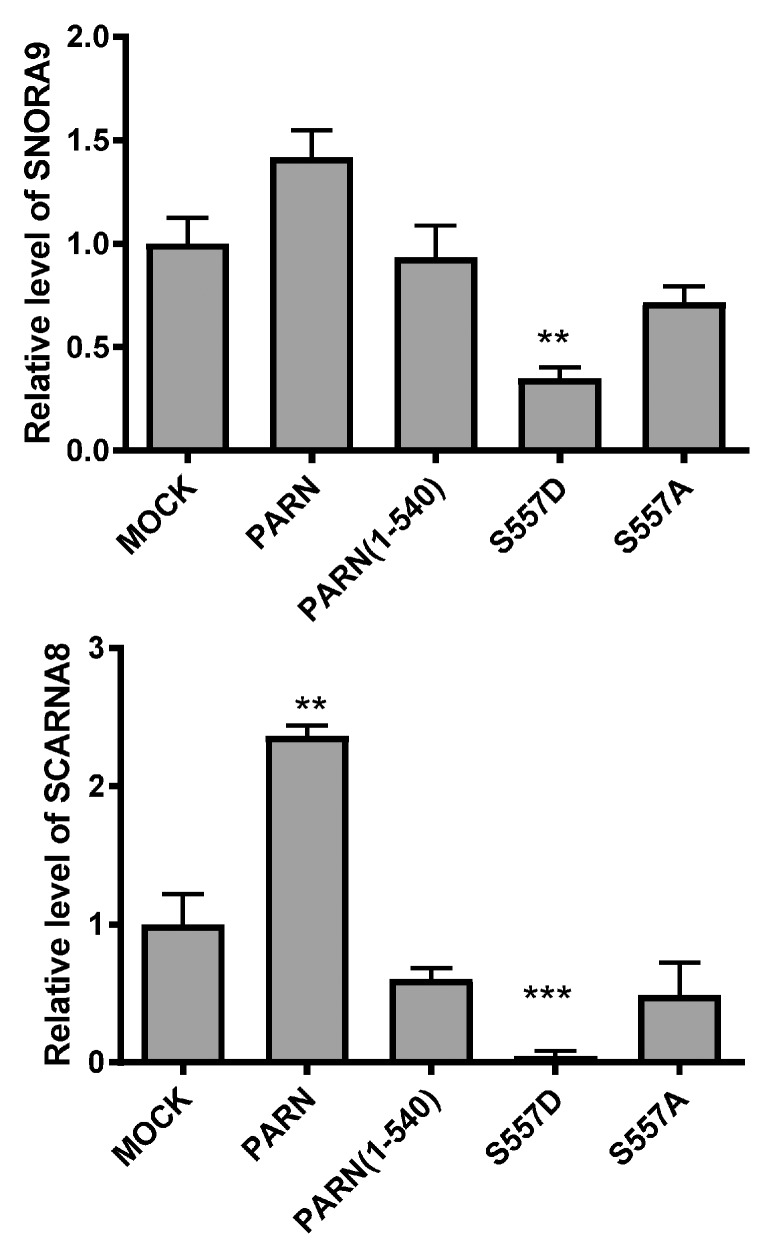
Effect of exogenously expressed wild type (WT) and mutated PARN on the RNA levels of SNORA9 and SCARNA8 in the HEK-293T cells under normal cell culturing conditions by qRT-PCR (*n* = 3). PPIA was used as the internal control. ** *p* < 0.01; *** *p* < 0.001.

In the nucleoli and Cajal bodies, PARN is involved in the 3′-trimming during snoRNA and scaRNA processing [13]. To evaluate the impact of the CTD on PARN function in the nucleus, SNORA9 and SCARNA8, two RNAs that have been shown to be substrates of PARN in the nucleus [13], were taken as examples. As shown in Figure 8, exogenously expressed PARN, but not PARN(1–540), slightly increased the RNA levels of SNORA9 and SCARNA8. Meanwhile, PARN-S557D, but not PARN-S557A, greatly reduced the levels of the two RNAs. The opposing actions of PARN-S557A and PARN-S557D suggested that DNA damage-induced phosphorylation of PARN switched its function towards the processing of small nuclear RNAs. Further research is needed to elucidate how phosphorylation modulates PARN function in the metabolism of small nuclear RNAs.

## 4. Discussion

PARN is a cytoplasm–nucleus shuttling multifunctional deadenylase that participates in diverse cellular processes [21]. Early biochemical and structural studies indicate that PARN has a strong preference to long poly(A) substrates, and therefore plays an important role in mRNA decay (reviewed in [21]). Particularly, PARN can direct the degradation of a couple of mRNAs encoding proteins vital to cell survival such as p53 [22,54], p21 [18], Gadd45α [23], nucleophosmin [55], c-fos and TNFα [56]. Besides regulating the poly(A) tail length of mRNAs, PARN is also capable of degrading the RNA poly(U) tail [57]. During the last decade, PARN has been shown to be critical to the maturation of various non-coding RNAs, including miRNAs [16,29,58,59], small nuclear non-coding RNAs [13,60], ribosomal RNAs [61] and telomerase RNAs [62,63,64,65]. Despite the increasingly recognized importance of the diverse functions of PARN, it remains unclear what parts of the sequence(s) direct the nuclear/nucleolar localization and which domains trigger protein–protein interactions. This paper answers these fundamental problems and provides insights in to the structural features and functional implications of the seldom studied PARN-CTD.

**Figure 9:**
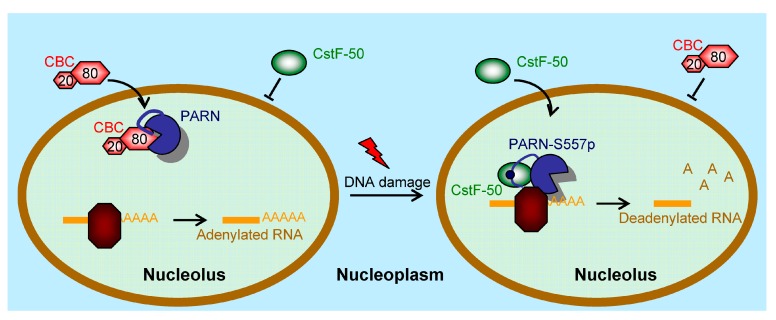
A working model of the function switch of PARN in the nucleoli during DNA damage response (DDR). Under normal conditions, PARN recruits the CBP80/CBC complex into the nucleoli and the deadenlyase activity is suppressed to facilitate RNA polyadenylation. During DDR, phosphorylation of PARN-S557 induces structure switch of the CTD to release CBP80 and expel the CBC complex from the nucleoli. Meanwhile, phosphorylated PARN prefers to bind with CstF-50 to enhance RNA deadenylation.

PARN shuttles between the nucleus and cytoplasm with nucleolar enrichment in common cell lines such as U2OS [13], CHO, HEK-293T and HeLa (this research). We further show that the NLS and NoLS in the CTD guide PARN to enter the nucleus and nucleoli, respectively. The strong localization signals of PARN-NLS and -NoLS can also greatly enhance the nuclear and nucleolar distributions of GFP fusion proteins (Figure 3). Neither mild DNA damaging conditions, induced by UV or HU treatment, nor overexpression of PARN binding partner CBP80/CstF-50 has any impact on PARN subcellular distribution. Therefore, the diverse subcellular distribution of PARN is probably gained by the dynamic shuttle between different cellular compartments. Under severe DNA damaging conditions, a translocation from nucleus to cytoplasm may occur, but the underlying mechanism remains unclear (Duan, Jiao, He and Yan, unpublished data). Nonetheless, our data support the idea that PARN is a nucleolus-retention protein under both normal and moderately stressed cellular conditions. It has been proposed that a distinct RNA-binding protein may recruit PARN to a subset of mRNAs in a specific workplace, and thereby ensure sophisticated regulation of the targeted RNAs [2]. However, the nucleolus-residing PARN can recruit binding proteins lacking NoLS in the nucleoli. More importantly, stress-induced post-translational modifications, such as phosphorylation, can switch the subset of binding partners, and thereafter switch its functions in the nucleoli (Figure 5 and Figure 7). A schematic summary of this function switch is presented in Figure 9. Under normal conditions, PARN recruits the CBP80/CBC complex into the nucleoli to suppress its deadenylase activity. Upon DNA damage, phosphorylated PARN expels CBP80, to discharge inhibition, and recruits CstF-50 into the nucleoli to activate the deadenylase activity. Our results clarify that the activation of PARN by CstF-50 is not caused by changes in expression level or modifications of CstF-50. Actually, the reactivated PARN can function in the absence of CstF-50 because the protein level of CstF-50 quickly drops down after DNA damage treatment. The reactivated PARN quickly reshapes the profile of matured small nuclear non-coding RNAs to respond to cellular stimuli and adapt to the changed living conditions (Figure 8).

The functional switch of PARN is achieved by a structural switch of the intrinsically disordered CTD, and S557 phosphorylation acts as the key modulator. Previously the CTD has been suggested to be intrinsically disordered by in silico analysis. Herein, we provide experimental evidence that PARN-CTD is dominated by disordered structures, but contains a considerable level of loosely packed local structures that can shield the only Trp fluorophore at the C-terminus (Figure 1). Post-translational modification is a common regulatory method to switch the folding status of intrinsically disordered proteins [52]. Unlike the well-defined unfolded to folded secondary structure transition in intrinsically disordered proteins, phosphorylation does not affect the secondary structures of PARN-CTD, but induces changes in the local structures and even the overall tertiary structure (Figure 6). The phosphorylation-mimic mutation S557D induces a microenvironmental change of the distal fluorophore W639, implying that the N- and C-termini are probably spatially adjacent in the unphosphorylated CTD. S557D disrupts the long-range interactions, exposes the W639 fluorophore to solvent and leads the loosely packed structure to an extensively disordered one. In the full length protein, the loosely packed structure of CTD restricts its interaction with other domains, while phosphorylation-induced extended CTD is capable of binding with the well-folded domains of PARN. It is worth noting that WT PARN-CTD contains two types of fluorophores, corresponding to the loosely packed structure and extended structure (Figure 1). Thus, PARN-CTD can adopt these two states with equal propensity and phosphorylation shifts the equilibrium between these two highly populated conformers to the extended one (Figure 6). Unfortunately, the expression yield of PARN-CTD is too low to perform high-resolution structural studies and further research is needed to verify our proposal.

Intrinsically disordered proteins are capable of being recognized by numerous binding partners [48]. The ability to adopt different conformers of the intrinsically disordered CTD endows PARN with the potency to bind with its diverse binding proteins. Besides CBP80 and CstF-50 studied here, our preliminary yeast-two-hybrid study comparing the potential interacting proteins of PARN, PARN(1–540) and PARN(541–639) indicates that the CTD mediates the interaction of a large number of PARN-binding proteins (He, Duan, Hu and Yan, unpublished data). Thus, it may be a general mechanism to switch PARN function by a post-modification-induced structural switch of the CTD, not only during DDR, but also for the other stress response processes or pathological conditions. Extensive phosphorylation of PARN has been found in cells subjected to serum deprivation [66]. Meanwhile, changes in PARN phosphorylation status have been observed in samples from patients with acute lymphoblastic leukemia and acute myeloid leukemia [67]. The switches of PARN structure and function can also occur in the nucleoplasm and cytoplasm. PARN phosphorylation by MK2 in the cytoplasm stabilizes Gadd45α mRNA, although the corresponding RNA-binding protein recruiting PARN to the Gadd45α mRNA remains unknown. Our unpublished data indicates that CTD phosphorylation can also modify the CTD-triggered endoplasmic reticulum (ER) -localization of PARN (Duan, Jiao, He and Yan, unpublished data). An unresolved problem is that, although the intrinsically disordered PARN-CTD undergoes a tertiary structure switch, the disordered–folding transition can also be induced by binding partners [48,68,69]. Further structural studies of the PARN-binding protein complex may provide more insight into the structure/function switch of the multifunctional deadenyalse PARN.

## Supplementary Materials

The following are available online at https://www.mdpi.com/2073-4409/8/8/836/s1, Figure S1: Effect of temperature, pH and K+ on PARN-CTD structural features monitored by far-UV CD and Trp fluorescence excited at 295 nm, Figure S2: K+ enhanced PARN-CTD Trp fluorescence in a concentration-dependent manner, implying that there is specific binding of K+ with PARN-CTD, Figure S3: Sequence alignment of the predicted NLS and NoLS, Figure S4: Representative profiles of flow cytometry analysis of cell apoptosis determined by Annexin V-FITC binding (horizontal) and PI exclusion (vertical), Figure S5: Western blot analysis of the protein levels of CstF-50 and p53 in untreated or UV-treated HEK-293T cells transfected with plasmids containing the full length or truncated PARN.
