# Supplementary material for: The Intrinsically Disordered C-Terminal Domain Triggers Nucleolar Localization and Function Switch of PARN in Response to DNA Damage
Source: Cells. 2019 Aug 5;8(8):836. doi: 10.3390/cells8080836 (PMC6721724; doi:10.3390/cells8080836)
Supplement: Supplementary file 1 [file cells-08-00836-s001.pdf]

Supplemental Materials

The intrinsically disordered C-terminal domain triggers nucleolar localization and function switch of PARN in response to DNA damage

Tian-Li Duan [†], Guang-Jun He [†], Li-Dan Hu and Yong-Bin Yan [*]

State Key Laboratory of Membrane Biology, School of Life Sciences, Tsinghua University, Beijing 100084, China

[†] These authors contributed equally to this work.

[*] **Correspondence:** Dr. Yong-Bin Yan, School of Life Sciences, Tsinghua University, Beijing 100084, China; Phone: +86-10-6278-3477; Fax: +86-10-6277-2245; E-mail: ybyan@tsinghua.edu.cn.

Running Title: Role of C-terminal domain in PARN function

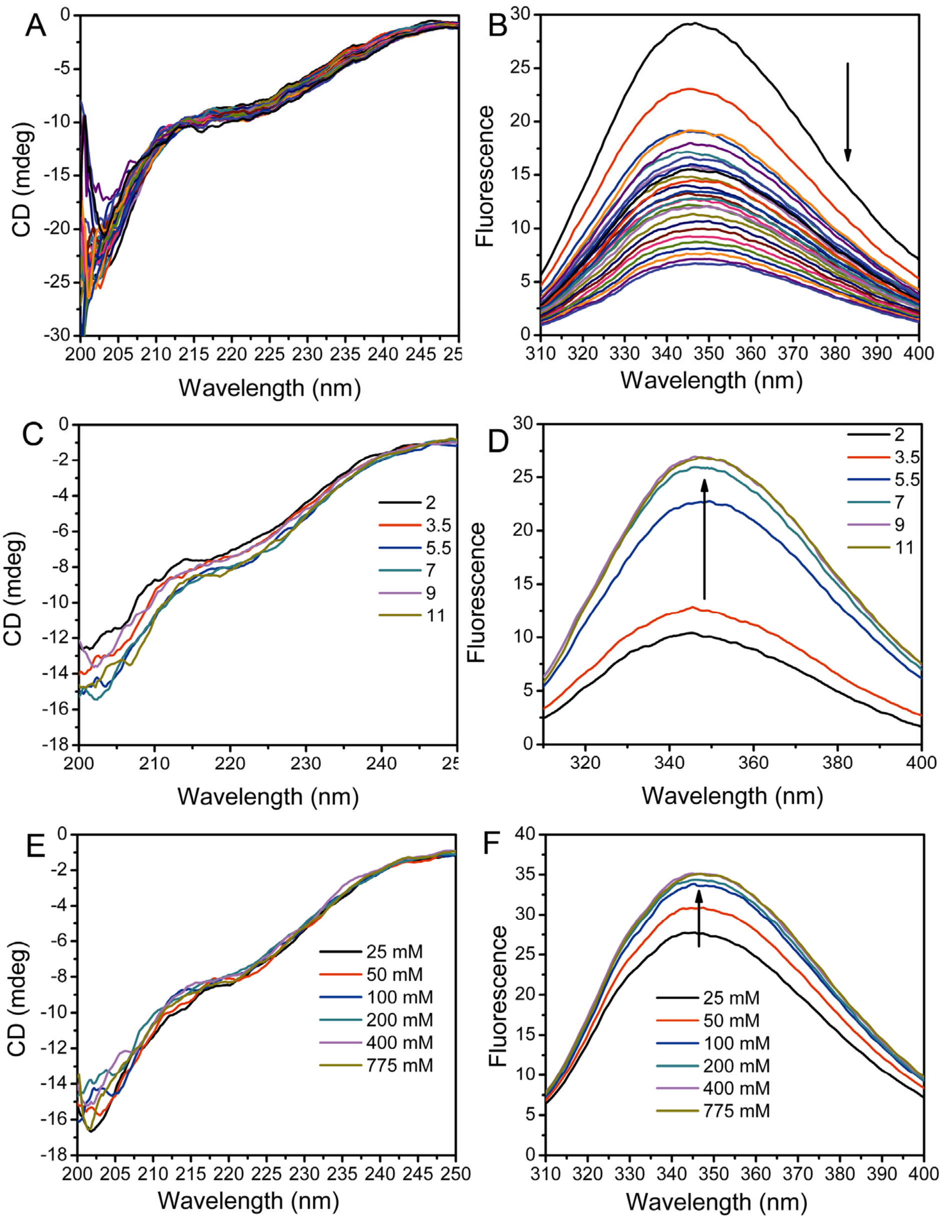

Figure S1. Effect of temperature (A and B), pH (C and D) and K^+ (E and F) on PARN-CTD structural features monitored by far-UV CD (A, C and E) and Trp fluorescence excited at 295 nm (B, D and F). Similar results were also obtained for Trp and Tyr fluorescence excited at 280 nm (data not shown). The factors did not influence PARN-CTD secondary and tertiary structures. Similar results were obtained for factors including divalent metals (Mg^{2+}, Mn^{2+} and Ca^{2+}), low concentration of ureal and macromolecular crowding reagent including dextran-70 and PEG-20000 (data not shown).

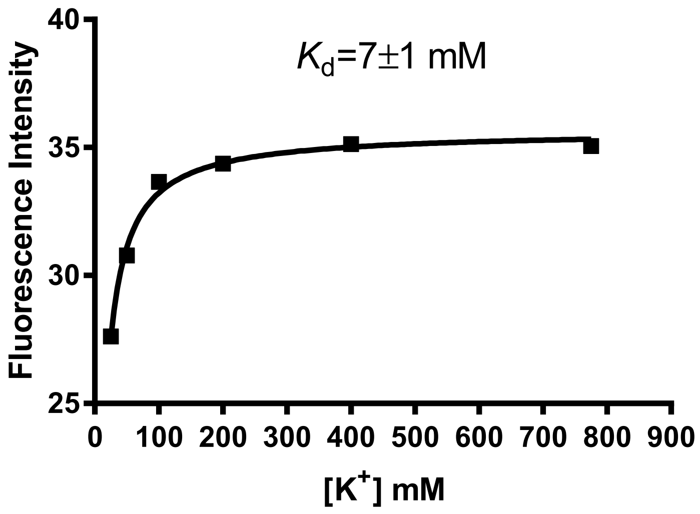

Figure S2. K^+ enhances PARN-CTD Trp fluorescence in a concentration-dependent manner, implying that there is specific binding of K^+ with PARN-CTD.

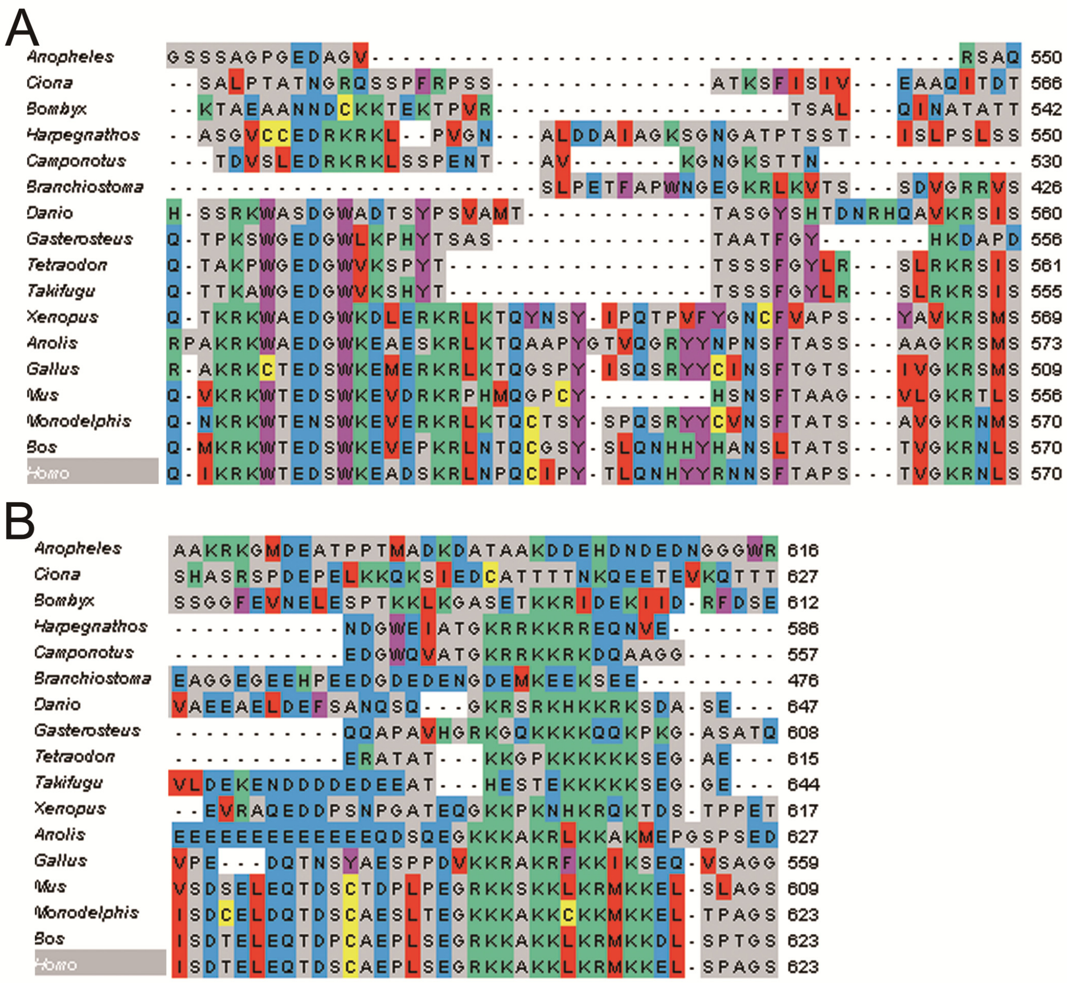

Figure S3. Sequence alignment of the predicted NLS (A) and NoLS (B) performed by the online software MAFFT (http://www.ebi.ac.uk/Tools/msa/mafft/). The sequences from bottom to top used for alignment are: *Homo sapiens* (NP_002573.1), *Bos taurus* (NP_001094588.1), *Monodelphis domestica* (XP_001375777.1), *Mus musculus* (NP_083037.1), *Gallus gallus* (NP_001025800.1), *Anolis carolinensis* (XP_003228705.1), *Xenopus (Silurana) tropicalis* (NP_001184102.1), *Takifugu rubripes* (XP_003972409.1), *Tetraodon nigroviridis* (CAF98128.1), *Gasterosteus aculeatus*, *Danio rerio* (NP_957382.1), *Branchiostoma floridae* (XP_002606119.1), *Camponotus floridanus* (EFN61018.1), *Harpegnathos saltator* (EFN75773.1), *Bombyx mori* (NP_001153677.1), *Ciona intestinalis* (XP_002129059.1), *Anopheles gambiae str. PEST* (XP_308433.4).

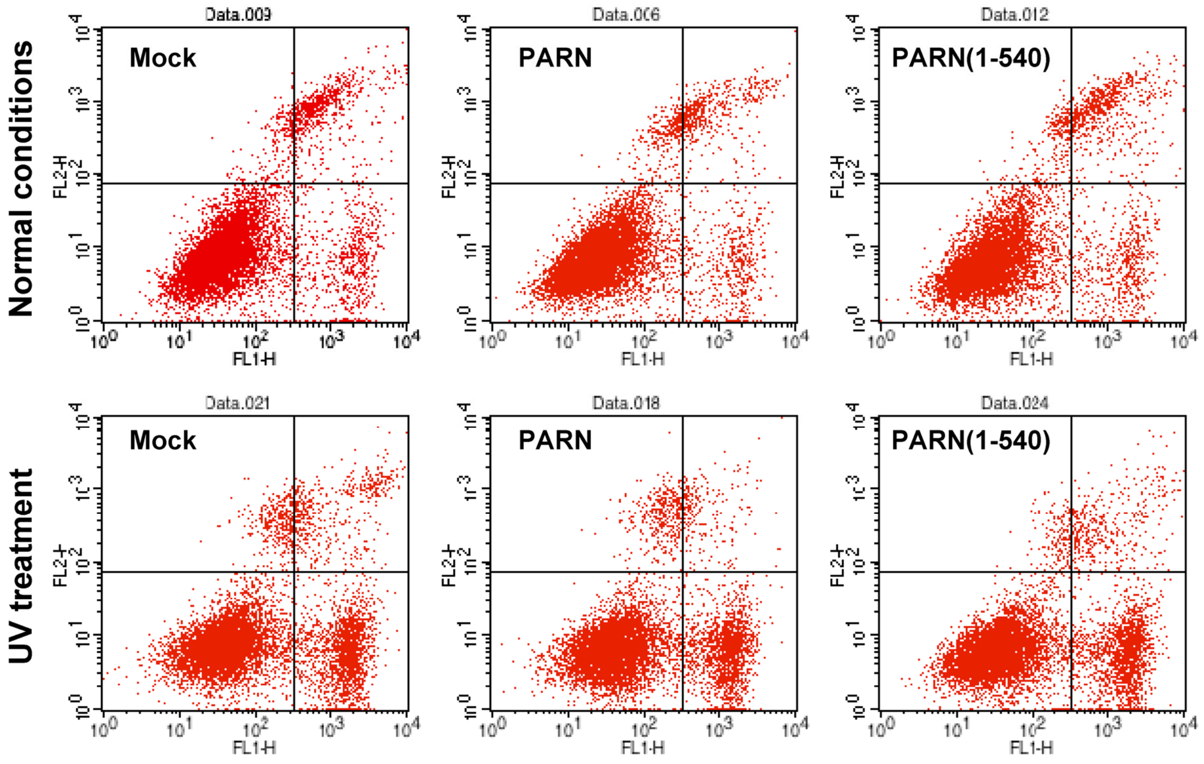

Figure S4. Representative profiles of flow cytometry analysis of cell apoptosis determined by Annexin V-FITC binding (horizontal) and PI exclusion (vertical). The HEK-293T cells were transfected by the empty vector (MOCK), Flag-PARN or Flag-PARN(1-540).

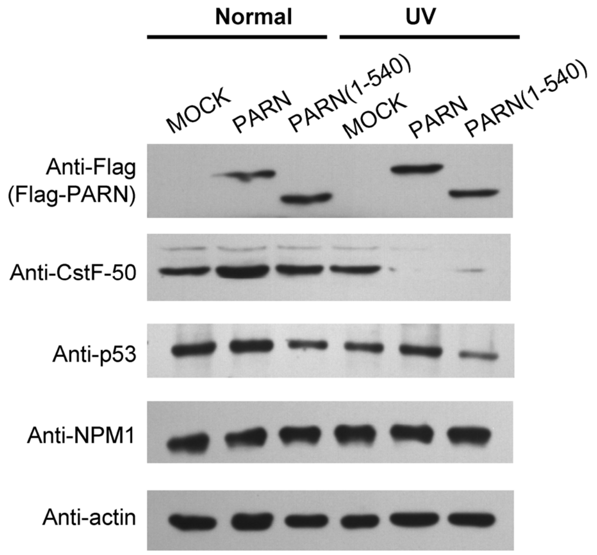

Figure S5. Western blot analysis of the protein levels of CstF-50 and p53 in untreated or UV-treated HEK-293T cells transfected with plasmids containing the full length or truncated PARN. NPM1 and actin were used as the controls.
